# Identifying plasma proteomic signatures from health to heart failure, across the ejection fraction spectrum

**DOI:** 10.1038/s41598-024-65667-0

**Published:** 2024-06-27

**Authors:** Karolina Andrzejczyk, Sabrina Abou Kamar, Anne-Mar van Ommen, Elisa Dal Canto, Teun B. Petersen, Gideon Valstar, K. Martijn Akkerhuis, Maarten Jan Cramer, Victor Umans, Frans H. Rutten, Arco Teske, Eric Boersma, Roxana Menken, Bas M. van Dalen, Leonard Hofstra, Marianne Verhaar, Jasper Brugts, Folkert Asselbergs, Hester den Ruijter, Isabella Kardys

**Affiliations:** 1 Department of Cardiology, Thorax Center, Cardiovascular Institute, Erasmus MC, University Medical Center Rotterdam, Rotterdam, The Netherlands; 2 Department of Cardiology, Franciscus Gasthuis & Vlietland, Rotterdam, The Netherlands; 3 Laboratory of Experimental Cardiology, University Medical Center Utrecht, Utrecht University, Utrecht, The Netherlands; 4 Department of General Practice & Nursing Science, Julius Center for Health Sciences and Primary Care, University Medical Center Utrecht, Utrecht University, Utrecht, The Netherlands; 5 Department of Biostatistics, Erasmus MC, University Medical Center Rotterdam, Rotterdam, The Netherlands; 6 Clinical Cardiology Department, University Medical Center Utrecht, Utrecht University, Utrecht, The Netherlands; 7 Department of Cardiology, Northwest Clinics, Alkmaar, the Netherlands; 8 Cardiology Centers of the Netherlands, Utrecht, The Netherlands; 9 Department of Nephrology and Hypertension, University Medical Center Utrecht, Utrecht University, Utrecht, The Netherlands

**Keywords:** Heart failure, HFpEF, HFrEF, Biomarkers, Proteomics, Cardiology, Medical research

## Abstract

Circulating proteins may provide insights into the varying biological mechanisms involved in heart failure (HF) with preserved ejection fraction (HFpEF) and reduced ejection fraction (HFrEF). We aimed to identify specific proteomic patterns for HF, by comparing proteomic profiles across the ejection fraction spectrum. We investigated 4210 circulating proteins in 739 patients with normal (Stage A/Healthy) or elevated (Stage B) filling pressures, HFpEF, or ischemic HFrEF (iHFrEF). We found 2122 differentially expressed proteins between iHFrEF-Stage A/Healthy, 1462 between iHFrEF–HFpEF and 52 between HFpEF-Stage A/Healthy. Of these 52 proteins, 50 were also found in iHFrEF vs. Stage A/Healthy, leaving SLITRK6 and NELL2 expressed in lower levels only in HFpEF. Moreover, 108 proteins, linked to regulation of cell fate commitment, differed only between iHFrEF–HFpEF. Proteomics across the HF spectrum reveals overlap in differentially expressed proteins compared to stage A/Healthy. Multiple proteins are unique for distinguishing iHFrEF from HFpEF, supporting the capacity of proteomics to discern between these conditions.

## Introduction

Heart failure (HF) is a multifaceted syndrome involving both cardiovascular and non-cardiovascular mechanisms. In clinical practice, HF is categorized based on left ventricular ejection fraction (LVEF), into two main types: heart failure with reduced ejection fraction (HFrEF) and heart failure with preserved ejection fraction (HFpEF)^1,2^. HFrEF and HFpEF are known to differ in the biological mechanisms and comorbidities involved^3^. Knowledge about circulating proteins may contribute to our further understanding of these differences. Proteins may exhibit specific up- or downregulation in one HF subtype compared to another, or compared to patients without HF, indicating which processes play a role in HF development. Moreover, there is a lack of HFpEF-specific biomarkers that could be utilized in clinical practice, which could potentially contribute to the diagnosis, monitoring and treatment of this condition.

Considering that systemic comorbidities are inherent to both HFrEF and HFpEF, the search for clinically significant biomarkers should not be confined solely to cardiac-specific proteins. Expanding the scope to include high-throughput aptamer-based proteomics, which enables the simultaneous measurement of thousands of proteins^4^, holds promise for further unravelling the pathophysiology and progression of cardiac diseases. This approach expands beyond those cardiovascular-related proteins traditionally studied in cardiac research, which further enhances its value in studying HF.

Thus far, two previous studies described proteomics profiles in relation to LVEF^5,6^. Adamo et al. investigated patients with HFrEF, HFpEF and HFmrEF and found unique variations in circulating proteins, which reflected distinct biological pathophysiologies of HF across LVEF. These included increase in proteins related to innate immunity and a decrease of proteins related to humoral immunity for HFpEF and increased growth factor signaling, ECM remodeling and angiogenesis for HFrEF^6^. The EXCEL trial compared a similar population of patients with type 2 diabetes and HFrEF, HFpEF and HFmrEF.

While studies in patients with HF are common, the ACC/AHA HF classification^2^ identifies stages that include (asymptomatic) patients at risk, and patients with cardiac abnormalities that are important in the trajectory towards HF. Proteomic investigations that differentiate between these stages could contribute to deeper understanding of the distinct mechanisms involved in the development of various types of HF. Moreover, although HFpEF affects women more often than men, and HFrEF vice versa, sex differences in HF subtype-specific proteomic profiles have not yet been addressed.

Against this background, we studied 4210 circulating proteins in men and women in different HF stages ranging from subjects at risk for HF but without clinical symptoms to patients with established HFrEF or HFpEF. This approach enables identification of differentially expressed proteins (DEPs) between individuals without signs of elevated filling pressures (patients at risk—Stage A, including healthy participants in our analysis), non-HF patients with elevated filling pressures (Stage B) and patients with ischemic HFrEF (iHFrEF) or HFpEF^2^.

## Results

### Baseline characteristics

The two cohorts that were examined consisted of a total of 739 individuals: 198 Stage A/Healthy, 326 with Stage B, 45 with HFpEF and 170 with iHFrEF. Baseline characteristics are shown in Table 1. Overall, mean (± Standard Deviation [SD]) age was 64 (± 10) years and 45% were men, but there were considerable differences in age- and sex distribution between the 4 groups. The mean (± SD) LVEF across the groups was as follows: Stage A/Healthy 68% (± 8), Stage B 67% (± 7), iHFrEF 28% (± 9) and HFpEF 66% (± 8). In general, patients with HF were older, and more often presented with diagnosed cardiac comorbidities and decreased kidney function, compared to Stage A/Healthy individuals as well as to Stage B. Individuals in the Stage B group also showed increased burden of cardiac diseases and risk factors compared to Stage A/Healthy (Table 1).

**Table 1:** Baseline characteristics.

Characteristic	Overall, n = 739^1^	Class A/Healthy, n = 198^1^	Class B, n = 326^1^	HFpEF, n = 45^1^	HFrEF, n = 170^1^
Age	64 (10)	58 (8)	65 (9)*^†^	71 (8)*	68 (10)*
Sex					
Female	403 (55%)	126 (64%)	218 (67%)	31 (69%)	28 (16%)^†^
Male	336 (45%)	72 (36%)	108 (33%)	14 (31%)	142 (84%)^†^
BMI	27.2 (4.3)	26.5 (3.8)	27.2 (4.4)^†^	29.3 (5.9)*	27.5 (3.9)*
LVEF	61 (17)	68 (8)	67 (7)	66 (8)	28 (9)*^†^
eGFR	79 (25)	86 (22)	82 (26)*^†^	73 (24)*	63 (21)*^†^
DM	99 (13%)	7 (3.5%)	28 (8.6%)*^†^	10 (22%)*	54 (32%)*^†^
Hypertension	415 (56%)	87 (44%)	210 (64%)*	31 (69%)*	87 (51%)*^†^
Dyslipidaemia	131 (18%)	8 (4%)	24 (7.4%)^†^	7 (16%)*	92 (54%)*^†^
AF	68 (9.2%)	2 (1%)	7 (2.1%)	2 (4.4%)*	57 (34%)*^†^
ARB	236 (32%)	12 (6.1%)	50 (15%)*	9 (20%)*	165 (97%)*^†^
Diuretics	246 (33%)	21 (11%)	62 (19%)*	14 (31%)*	149 (88%)*^†^
Beta-blockers	237 (32%)	18 (9.1%)	53 (16%)*	14 (31%)*	152 (90%)*^†^
MRA	124 (17%)	2 (1%)	3 (0.9%)	0 (0%)	119 (70%)*^†^
CCB	220 (30%)	15 (7.6%)	35 (11%)	6 (13%)	164 (97%)*^†^
Anticoagulants	161 (22%)	5 (2.5%)	15 (4.6%)	7 (16%)*	134 (79%)*^†^
Statins	276 (37%)	24 (12%)	89 (27%)*	18 (40%)*	145 (80%)*^†^

^1^Mean, (SD); n (%).

*p-value < 0.05, versus class A/Healthy; Welch t-test, Chi-squared test; Fisher's exact test, Mann–Whitney *U* test, where appropriate.

^†^p-value < 0.05, versus HFpEF, Welch t-test, Chi-squared test; Fisher's exact test, Mann–Whitney *U* test, where appropriate.

*BMI* Body mass index, *LVEF* Left ventricular ejection fraction, *AF* Atrial fibrillation, *DM* Diabetes mellitus, *ARB* Angiotensin II receptor blockers, *MRA* Mineralocorticoid receptor antagonist, *CCB* Calcium channel blocker.

**Figure 1:**
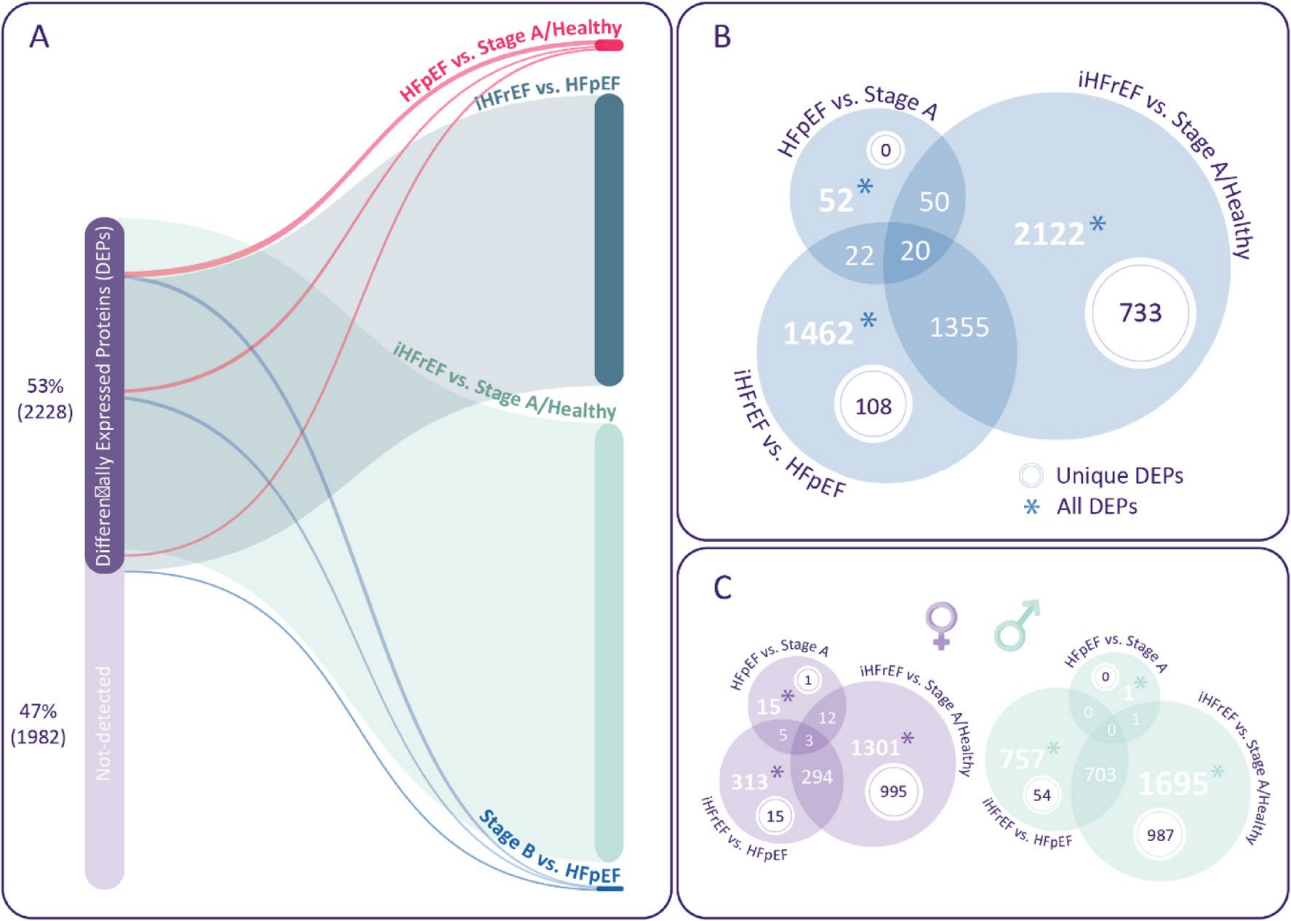
(**A**) Modified parallel set diagram showing the proportion and overlap of DEPs found between the groups in the full population. (**B**) Euler diagram of shared and unique DEPs found between the groups in the full population. Unique DEPs are defined as DEPs found only between two groups, but not between other groups. (**C**) Euler diagrams of DEPs found in the analyses in women and men.

In total, the plasma measurement resulted in 4210 unique proteins for analysis. An overview of the number of proteins that showed different expression compared to the reference group (which consecutively consisted of Stage A/Healthy and HFpEF), is shown in Table 2. A graphical representation of detected DEPs is shown in Fig. 1. A summary of the findings is provided in Supplementary Fig. S1.

**Table 2:** Number of significantly associated differentially expressed proteins (DEPs) versus a reference group (stage A/healthy or HFpEF).

Number of associated proteins	All	Women	Men
Reference: stage A/healthy			
Stage B	0	0	0
HFpEF	52	15	1
iHFrEF	2122	1301	1695
Reference: HFpEF			
Stage B	27	5	3
iHFrEF	1462	313	757

### HFpEF vs. stage A/healthy

In total, 52 DEPs were found between the HFpEF group vs. Stage A/Healthy. Out of the 52 proteins, 18 were expressed in higher levels in HFpEF, with NT-proBNP (NPPB) showing the strongest association. Among the 34 DEPs that were expressed in lower levels in HFpEF compared to Stage A, NELL2 showed the strongest association.

Two of the 52 proteins that were different in HFpEF versus Stage A/Healthy are predominantly expressed in the heart (FABP3, NPPB)^7^. However, 50 out of 52 of the detected DEPs were not cardiac specific. Several of these proteins are predominantly expressed in specific tissues: adipose tissue (PXDN, RBP7), kidney (HAVCR1), lungs (ACVRL1) or liver (GDF2, ASGR1, EPO, IGFALS), suggesting importance of these organs in HFpEF development^7^. According to Gene Ontology terms, 8 out of the 52 proteins were significantly linked to growth factor binding (ACVRL1, IGFALS, IGFBP3, NTRK3, NTRK2, EGFR, IL1RN, PXDN); and 2 to interleukin-1 receptor antagonist activity (PXDN, IL1RN).

**Figure 2:**
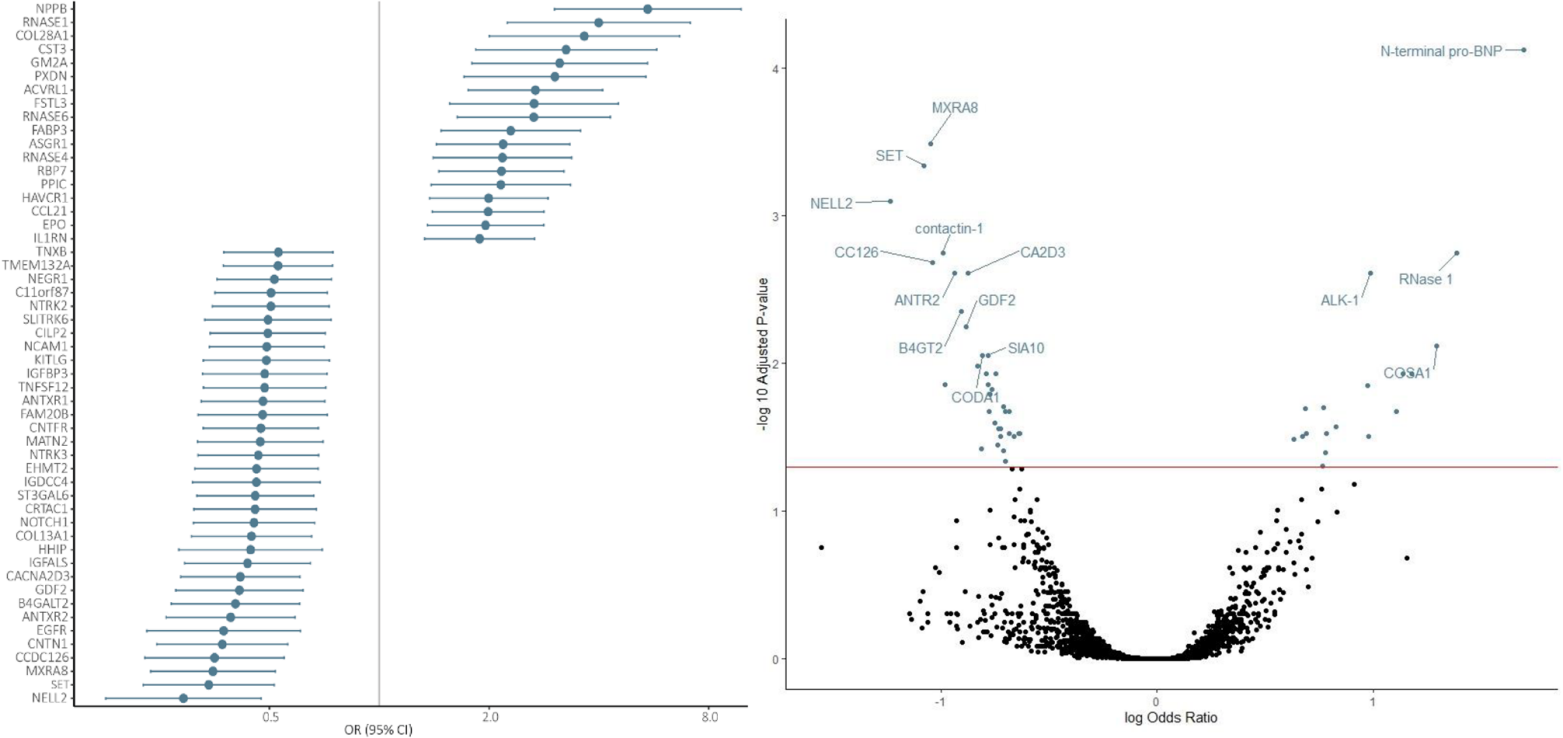
DEPs found in HFpEF vs. Stage A/Healthy. On the left side of the figure we plotted the single-protein associations (adjusted for age and sex) with HFpEF compared to Stage A/Healthy (reference group), resulting from the multinominal regression analysis. The HF group was used as the dependent variable and the protein level as independent variable. All 52 that showed statistically significant associations after adjustment for multiple testing are presented. X-axis is on the log2 scale. The right side of the figure presents a full overview of the comparison of HFpEF with Stage A/Healthy (reference group), resulting from the multinominal regression analysis, including the 52 proteins that showed statistically significant associations, with the top 15 labelled. The y-axis shows the – log 10 adjusted p-value, and the x-axis shows the (natural) log odds ratio.

All detected DEPs between HFpEF and Stage A/Healthy are shown in Fig. 2. The full list of DEPs found in HFpEF vs. Stage A/Healthy with their respective ORs and 95% confidence intervals, is shown in Supplementary Table S1.

### iHFrEF vs. stage A/healthy

For iHFrEF, we found 2122 DEPs compared to Stage A/Healthy. Among these proteins, 50 DEPs were also detected between HFpEF vs. Stage A/Healthy, leaving only 2 biomarkers being uniquely downregulated for HFpEF, namely SLITRK6 (SLIT And NTRK Like Family Member 6) and NELL2 (Neural EGFL Like 2). Of the 50 DEPs that emerged both for HFpEF and iHFrEF, 49 showed the same direction of protein expression. One protein showed opposite direction of expression, namely Matrilin-2 (MATN2). The levels were lower in HFpEF and higher in iHFrEF, vs. Stage A/Healthy. Moreover, the majority of these shared DEPs showed the strongest differences also between iHFrEF and Stage A/Healthy (e.g., NPPB, COL28A1, FAPB3, PXDN, GM2A, CST3). On the contrary, multiple coagulation factors showed lower levels in iHFrEF (e.g., F2, F9, F10). Among others, the lowest levels were detected for: AGT (Angiotensinogen) and EGFR (Epidermal growth factor receptor). Out of the detected 2122 DEPs, 733 were detected only between iHFrEF vs. Stage A/Healthy, but not between other groups.

Protein Set Enrichment Analysis showed that the biological processes in iHFrEF were associated with nucleobase, nucleoside, nucleotide and nucleic acid metabolic processes of biosynthesis, metabolism and binding. Molecular pathways in iHFrEF were related to cell proliferation, differentiation and regeneration. Visualization of Protein Set Enrichment Analysis is shown in Fig. 2.

**Figure 3:**
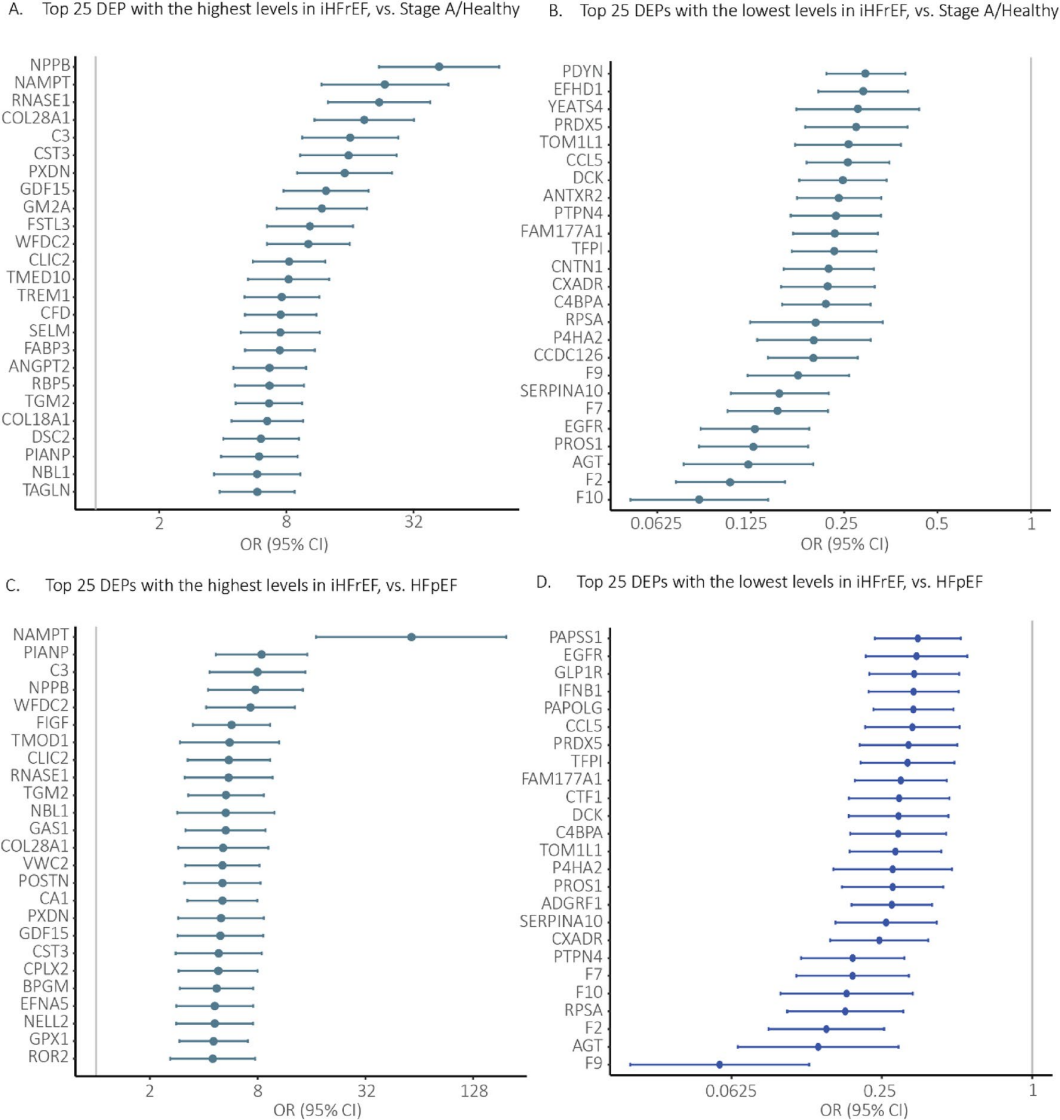
Top 25 DEPs with the highest and the lowest levels found in iHFrEF vs. Stage A/Healthy and iHFrEF vs. HFpEF in full population. In this figure we plotted the single-protein associations (adjusted for age and sex) with iHFrEF compared to Stage A/Healthy as the reference group (Fig. S1A,B) and HFpEF as the reference group (Fig. S1C,D), resulting from the multinominal regression analysis. The HF group was used as the dependent variable and the protein level as independent variable. Top 25 DEPs the highest and lowest levels that showed statistically significant associations after adjustment for multiple testing are presented. X-axis is on the log2 scale.

**Figure 4:**
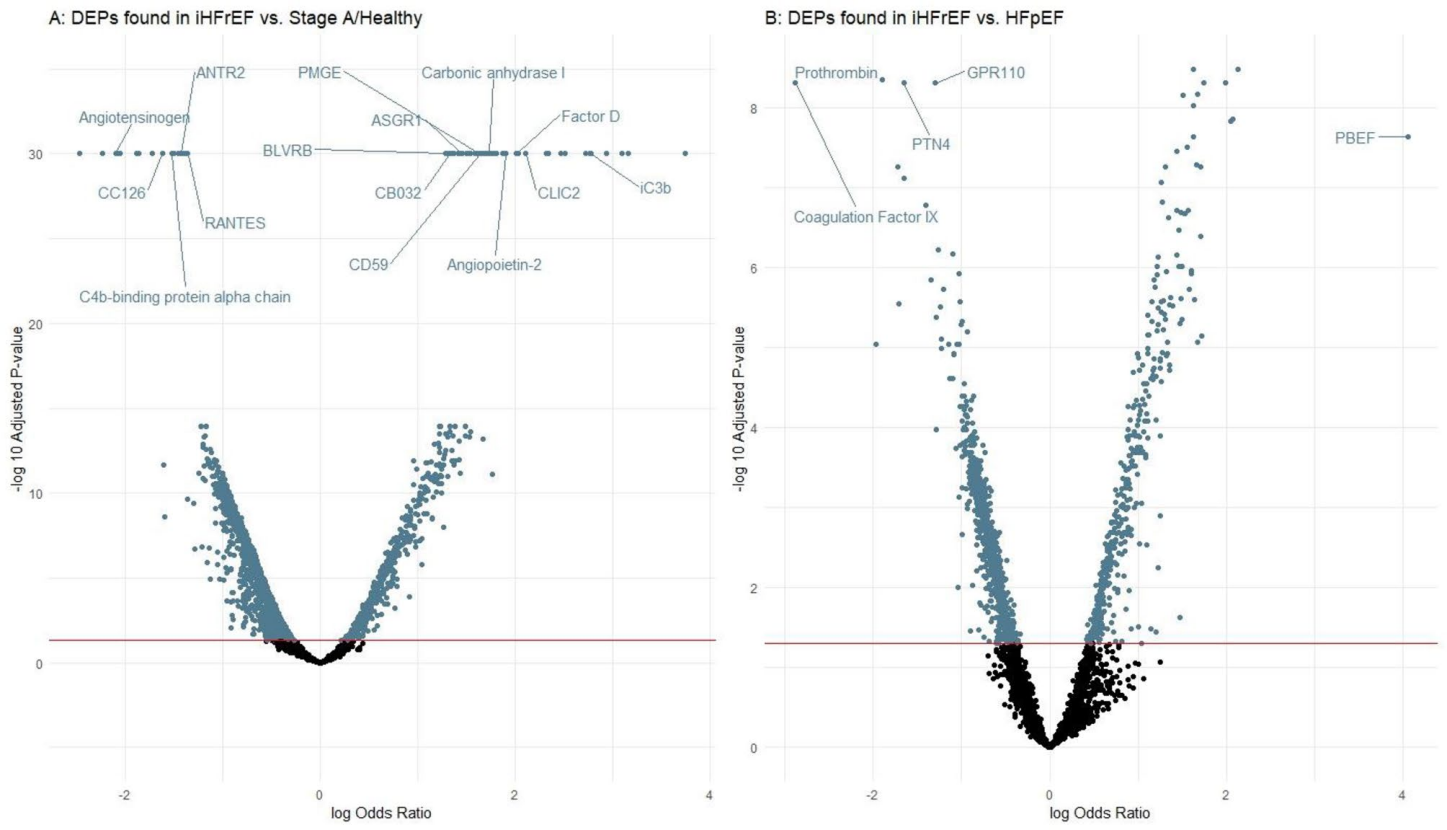
Overview of proteins associated with iHFrEF vs. Stage A/Healthy and iHFrEF vs. HFpEF in full population. This figure presents a full overview of the comparison of iHFrEF with Stage A/Healthy and HFpEF, respectively, resulting from the multinominal regression analysis, including all proteins that showed statistically significant associations, with the top 15 labelled. The y-axis shows the – log 10 adjusted p-value. The y-axis is truncated at a value of 30, and values exceeding 30 are represented at y = 30. The x-axis shows the (natural) log odds ratio.

The top 25 DEPs with the highest and the lowest expression levels between iHFrEF vs. Stage A/Healthy are shown in Fig. 3. An overview of all proteins in presented in Fig. 4. The full list of DEPs found between iHFrEF vs. Stage A/Healthy with their respective ORs and 95% confidence intervals is shown in Supplementary Table S2. The list of the DEPs uniquely found between iHFrEF vs. Stage A/Healthy is shown in Supplementary Table S3.

### iHFrEF vs. HFpEF

We detected 1462 DEPs in the plasma of patients with iHFrEF compared to HFpEF. The protein that showed the largest difference in level between iHFrEF and HFpEF was NAMPT (Nicotinamide phosphoribosyltransferase). Many of the DEPs with the highest levels for iHFrEF compared to HFpEF also showed high levels in iHFrEF vs. Stage A/Healthy and HFpEF vs. Stage A/Healthy (e.g., NPPB, COL28A1, RNASE1). Among the most decreased DEPs, proteins like AGT and EGFR and multiple coagulation factors were found (i.e. F2, F7, F9, F10).

Furthermore, 1356 DEPs detected between iHFrEF vs. HFpEF were also found between iHFrEF vs. Stage A/Healthy or HFpEF vs. Stage A/Healthy, leaving 108 DEPs found only between HFpEF and iHFrEF. To understand the role of these proteins in molecular pathways, we used Gene Ontology, and found that 5 of these proteins are involved in regulation of cell fate commitment (IL12RB1, BMP4, FGF2, SPDEF, FGFR1), particularly mesodermal cell fate commitment. Protein Set Enrichment Analysis showed remarkably more biological processes and pathways for which the subset of DEPs found between iHFrEF vs. HFpEF was enriched, than for the DEPs between iHFrEF vs. Stage A/Healthy. These processes included nucleobase, nucleoside, nucleotide and nucleic acid metabolic processes of biosynthesis, metabolism and binding. Molecular pathways in HFpEF were related to inflammation and metabolism.

**Figure 5:**
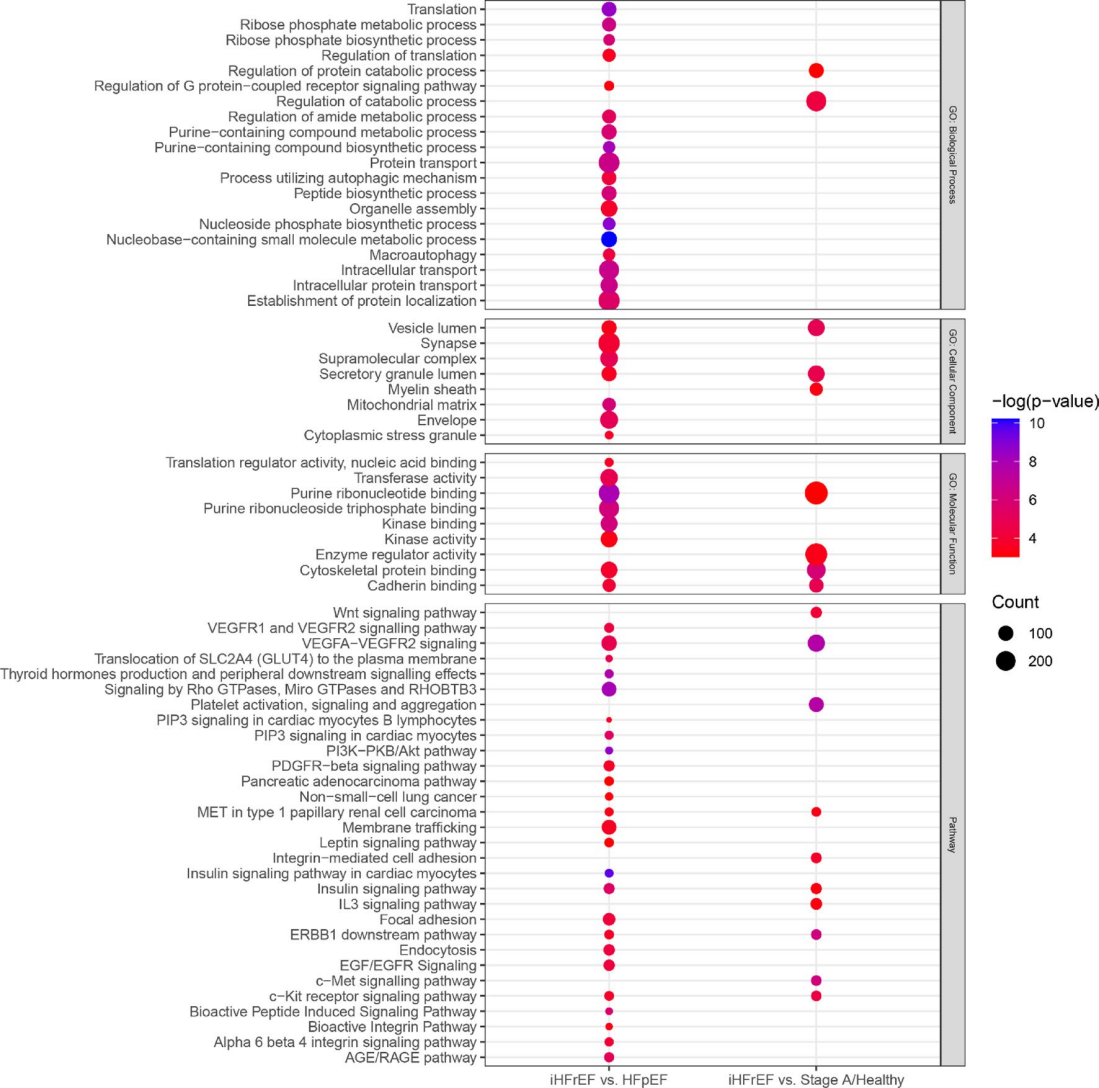
Protein set enrichment analysis of all significant differentially associated proteins (DEPs) between iHFrEF vs. HFpEF and iHFrEF vs Stage A/Healthy. In this figure the colour of the dot represents the magnitude of the p-value. The size (“Count”) represents how many of the included proteins are present in the mechanism or process.

Top 25 DEPs showing the largest differences in levels (both higher and lower levels) between iHFrEF and HFpEF are shown in Fig. 3. The full list of DEPs found between iHFrEF vs. HFpEF with their respective odds ratios and 95% confidence intervals is shown in Supplementary Table S2. The list of the DEPs uniquely found between iHFrEF vs. HFpEF is shown in Supplementary Table S3. Visualization of Protein Set Enrichment Analysis is shown in Fig. 5.

### Stage B vs. HFpEF

To identify proteins involved in the advanced stage of HFpEF, we investigated DEPs between Stage B (pre-HF) and HFpEF. We identified 27 DEPs between these stages. Out of these 27 proteins, 24 were also significantly different between HFpEF vs. Stage A/Healthy, showing the same direction of expression, but with stronger effect. CD320, GALNT1 and TFF2 were not found in HFpEF vs Stage A/Healthy. CD320 was the only biomarker found uniquely between Stage B and HFpEF but not between other groups. The full list of DEPs is shown in Supplementary Table S2.

### Differentially expressed proteins and sex differences

We detected 15 DEPs for HFpEF vs. Stage A/Healthy (3 with lower levels and 12 with higher levels in Stage A/Healthy) in women. None of the genes encoding these proteins was located on chromosome X. All DEPs were also present in the results derived from the full population of men and women. Only 1 DEP was found in men for HFpEF vs. Stage A/Healthy, namely ADM2 (Adrenomedullin 2). This protein was not present when analysing the full population (both men and women).

When comparing iHFrEF with Stage A/Healthy, 1301 and 1695 DEPs were found in women and men, respectively. When comparing iHFrEF with HFpEF, there were 313 and 757 DEPs in women and men, respectively. In men, NAMPT was the most expressed DEP for iHFrEF vs Stage A and for iHFrEF vs. HFpEF. In women, NPPB was the most expressed biomarker in iHFrEF vs. Stage A, but NAMPT for iHFrEF vs. HFpEF. Similarly to the analysis on the full population, among the DEPs with the lowest levels, AGT, EGFR and multiple coagulation factors were found (i.e. F2, F7, F9) between iHFrEF vs. Stage A/Healthy and iHFrEF vs. HFpEF.

All DEPs found in HFpEF vs. Stage A/Healthy in women are shown in Supplementary Fig. S1. Top 25 DEPs with the highest and the lowest levels between iHFrEF and Stage A/Healthy and between iHFrEF and HFpEF in women and men are shown in Supplementary Fig. S2. The whole list of DEPs found between the groups for men and women, with their respective ORs and 95% confidence intervals is available in Supplementary Table S4. Visualization of Protein Set Enrichment Analysis is shown in Supplementary Fig. S3.

## Discussion

In this study of 739 patients with a wide range of ejection fraction we investigated shared and unique DEPs between several groups. We found that, compared to Stage A/Healthy, a larger number of DEPs is present for iHFrEF than for HFpEF, and that the DEPs for iHFrEF include nearly all DEPs for HFpEF. Conversely, there are many DEPs between iHFrEF and HFpEF. Investigating DEPs that were found uniquely between two specific groups allowed us to identify proteins being specifically involved in development of a particular type of HF. We found several DEPs only between iHFrEF and HFpEF and only between iHFrEF and Stage A/Healthy, which underscores the capacity of proteomics to discern HFpEF from iHFrEF.

An important strength of our study is the inclusion of a broad spectrum of patients, encompassing various ranges of LVEF, as well as individuals without HF (with and without cardiac abnormalities). To the best of our knowledge, no previous studies have examined this many groups simultaneously, employing such a comprehensive proteomics panel, which adds to the novelty and strength of our findings^5,6,8^. Zannad et al. examined the effect of SGLT2 inhibitors on circulating proteins in patients with HFrEF and HFpEF, and found changes in levels of proteins linked to the heart and kidney that promote autophagic flux, nutrient deprivation signaling and transmembrane sodium transport^8^. Two studies also examined the unique proteomic signatures for patients with HFrEF and HFpEF. In the study by Adamo et al., differences were found in the levels of circulating proteins among patients with HF across the spectrum of LVEF, reflecting diverse underlying pathophysiological mechanisms not fully delineated by EF categorizations^6^. In the study by Peters et al., it was shown that across the LVEF spectrum in patients with type 2 diabetes and HF, levels of the majority of proteins demonstrated concordance between HFmrEF and HFpEF, but there were higher levels in HFrEF^5^. Our study confirms these findings, but also extends them by including healthy participants or individuals at risk of heart failure. These previous investigations primarily focused on patients with HF and did not encompass healthy participants or individuals at risk, while we were able to identify circulating proteins that are shared or unique to different HF groups.

Finding specific biomarkers for HFpEF was particularly warranted because of the current lack thereof in clinical practice. We found 52 DEPs between individuals with Stage A/Healthy and HFpEF. A few of these 52 proteins were previously implicated in HFpEF (i.e., FABP3, CYS3, FSTL3), but none of them is considered to be up- or downregulated specifically for this type of HF^9,10^. DEPs implicated by our results were associated with cardiac traits such as cardiac remodelling (EGFR, NOTCH1) and cardiomyocyte hypertrophy (EGFR)^11,12^. Some proteins play a role in the systemic response to inflammation (FSTL3), fibrosis and extracellular matrix formation (PXDN), as well as renal function (IGFBP-3, CST3, EPO), obesity (FSTL3), diabetes (IGFBP-3) and lung diseases (GM2A)^9,13–18^. Two proteins (ACVRL1, GDF2) are known to be associated with pulmonary hypertension and hereditary haemorrhagic telangiectasia (also known as Osler–Weber–Rendu disease)^19^. The phenotype of Osler–Weber–Rendau disease includes high output cardiac failure, often being recognized as HFpEF given the normal systolic function.

Two significant biological processes were identified with gene enrichment analyses of the 52 DEP2s, namely growth factor binding and interleukin-1 receptor antagonist activity.

Growth factor binding processes has been identified as relevant pathways for both HFpEF as HFrEF^20^. The peptide hormone insulin-like growth factor 1 (IGF-1) plays a role in regulating cell proliferation, differentiation, metabolism, and survival across various tissues^21^. By upregulating the IGF1-PI3K-Akt pathway, IGF-1 exhibits cardioprotective effects, improves cardiomyopathy, and modulates cellular processes involved in short-term ventricular remodeling of the infarcted myocardium^22^. Although lower levels of IGF have consistently been associated with worse outcomes, the effect of the IGF axis in HFpEF and disease progression is complex^22^. On the one hand, IGF-1 deficiency may promote the development of atherosclerotic cardiovascular disease by impairing the nitric oxide pathway. Conversely, a decrease in IGF signaling and subsequent activation of the PI3K/Akt pathway may attenuate senescence-related cardiac hypertrophy, interstitial fibrosis, inflammation, and oxidative stress, offering protective effects in HFpEF^22^.

The other pathway found to be associated with HFpEF was the interleukin-1 receptor antagonist activity. HF and especially HFpEF is known to be associated with chronic inflammation and there are multiple studies linking interleukin-1 hyperactivity with HF. The ability of IL-1 to directly modulate cardiomyocyte contractility has been long recognized as well^23,24^. Furthermore, CRP often regarded as a marker of interleukin-1 activity, exhibits positive correlations with left ventricular end-diastolic pressure, peak oxygen consumption, and ventilatory efficiency^23,24^. Studies on interleukin-1 blockage in HFpEF are scarce. In two small trials involving patients with HFpEF and diastolic dysfunction, interleukin-1 blockage demonstrated a modest, yet noteworthy enhancement in peak oxygen consumption, treadmill exercise duration, along with reductions in NTproBNP levels^23–25^. Additional studies are warranted to further elucidate these findings.

Out of 52 DEPs, 50 were also found when comparing iHFrEF with Stage A/Healthy. Only 2 proteins (SLITRK6—SLIT And NTRK Like Family Member 6 and NELL2—Neural EGFL Like) were expressed in significantly lower levels in HFpEF vs. Stage A/Healthy and in HFpEF vs. iHFrEF, but not detected between iHFrEF vs. Stage A/Healthy, indicating their specificity for HFpEF. SLITRK6 has been reported to be associated with COPD and upregulated in response to ischemic injury in the infarct zone and adverse cardiac remodeling following myocardial infarction^26^. Since ischemia is also common in HFpEF^27^, downregulation of SLITRK6 may indicate possible usability of SLITRK6 as a biomarker for HFpEF, regardless of presence of ischemia. NELL2 has been reported to be downregulated in HFpEF vs. HFrEF^28^, but its role in the development of HF remains unknown. Thus, the clinical relevance of these biomarkers still needs to be established. Initially, extensive pre-clinical research, and validation studies in large cohorts, are necessary to investigate and validate the two biomarkers. Subsequently, laboratory assays fit for clinical use could potentially be developed, and validated before they can be implemented in clinical practice. Among other DEPs found between HFpEF and Stage A/Healthy, one protein showed an opposite direction of expression in HFpEF and iHFrEF, namely Matrilin-2 (MATN2). MATN2 has been reported to be upregulated in HF, but reports regarding HFpEF in particular are lacking^29^. The specific function of MATN2 is not yet determined, but it may play a role in extracellular matrix formation and inflammation^30^. Both MATN2 and NELL2 have been documented to physically interact with each other^31^. Another noticeable finding was high expression of Nicotinamide phosphoribosyltransferase (NAMPT) in iHFrEF. Since upregulation of NAMPT is known to be present in ischemia, this may in part explain our findings^32^.

We also found multiple coagulation factors among the proteins with low expression levels in iHFrEF vs. HFpEF and Stage A/Healthy. Patients treated with VKA present lower levels of coagulation factors F2, F7, F9, F10, proteins C and protein S^33^. Significantly decreased coagulation factors between iHFrEF and others groups may partially be explained by the use of vitamin K antagonists (VKA) in a high percentage of the iHFrEF patients compared to the other groups.

Differential expression of proteins reported to be biomarkers in patients with HFpEF, such as Galectin-3 or Growth/differentiation factor 15 (GDF15)^34,35^ were detected only between HFpEF vs. iHFrEF and/or iHFrEF vs. Stage A/Healthy, but not between HFpEF vs. Stage A/Healthy. Both biomarkers are reported to be associated with multiple cardiac, but also non-cardiac traits such as: smoking, diabetes, blood pressure, serum lipids, BMI and kidney function (Galectin-3)^36,37^; cancer, type 2 diabetes (GDF15)^38^. Possibly the presence of these factors, which are frequently encountered in the HFpEF population, may contribute to the reduced specificity of these biomarkers in our analysis.

To elucidate the contribution of the identified proteins in the development of HF, we conducted Protein Set Enrichment Analysis. The analyses showed that, whereas pathways enriched in iHFrEF vs. Stage A/Healthy play a role in cardiac cell proliferation, differentiation and cardiac regeneration (i.e. Wtn signaling pathway)^39^, processes discriminating iHFrEF from HFpEF are involved in cardiac hypertrophy (PI3K-PKB/Akt signaling) and relaxation (EGF/EGFR signaling)^40^. Other pathways found for iHFrEF are related to oxidative stress control (c-Met signaling)^41^ and activation of cardioprotective mechanisms (IL3 signaling)^42^, while for HFpEF mechanisms encompassed response to inflammation (EGF/EGFR signaling)^11^ and glucose and lipid metabolism (Leptin signaling)^43^. Conversely, shared pathways included insulin signaling and angiogenesis (VEGFA-VEGFR2 signaling)^44^, suggesting the importance of these mechanisms, regardless of the HF etiology. Moreover, these findings point towards potential therapeutic avenues for mitigating the effects of oxidative stress and enhancing cardiac protection in iHFrEF.

Furthermore, the sex-based subanalysis showed remarkable differences in number of detected DEPs, as well as molecular processes and pathways involved in HF development, between men and women. Notably, more DEPs were detected for iHFrEF in men than in women. On the contrary, only one DEP was found for HFpEF vs. Stage A/Healthy in men, namely Adrenomedullin 2 (ADM2). ADM2 has previously been implicated in HFpEF and pulmonary hypertension^45^. In women, two other DEPs (ACVRL1, GDF2) have been linked to pulmonary hypertension^46^. The differential protein profiles observed in men and women suggest a significant role of pulmonary vascular disease in HF, albeit through distinct mechanistic pathways. Likewise, more molecular mechanisms exhibited enrichment for iHFrEF compared to HFpEF or Stage A/Healthy in men than in women. In men these processes play a role in, among others, cardiac proliferation (PDGFR—beta signaling pathway)^47^, cardiac remodeling and hypertrophy (Rho signaling, RAF signaling)^48,49^, angiogenesis (VEGFA-VEGFR2 signaling)^44^. Conversely, in women, a distinct emphasis on the involvement of the coagulation cascade was evident in iHFrEF development. The disparities found between women and men could be linked to the influence of sex hormones or receptors^50^, the presence of sex-specific genes^50,51^ and differences in the cardiac structure and function^52^. Moreover, women tend to have a higher expression of proinflammatory genes and higher levels of inflammatory cytokines. However, the precise mechanisms underlying these differences remain incompletely understood. In the future, these sex-based differences in proteomic signatures could impact clinical practice. Sex-specific diagnostic strategies could potentially play a role, such as employing specific biomarkers and different cutoffs for each of the sexes. Furthermore, integrating sex-specific factors into risk assessment models might assist in determining appropriate treatment approaches, thus customizing heart failure management based on sex^52^.

Altogether, we found a higher number of DEPs between iHFrEF and Stage A/Healthy and between iHFrEF and HFpEF, especially among male participants, compared to HFpEF and Stage A/Healthy. That suggests that pathological mechanisms involved in development of iHFrEF are notably distinct from those involved in HFpEF. These findings also align with the notion that identifying specific biomarkers exclusively associated with HFpEF presents a considerable challenge. The heterogeneity of HFpEF in the absence of universally accepted definitions that can distinguish between the various HFpEF phenotypes contribute to this. Our study adds to the growing body of evidence emphasizing the urgent need for more refined practical definitions of HFpEF phenotypes to facilitate the discovery of novel biomarkers.

## Limitations

Some limitations should be acknowledged. First, we used data from 2 cohorts for this investigation, which could entail variability stemming from differences in patient demographics, clinical characteristics, and data collection protocols. Furthermore, the cohorts may not fully represent the diversity present within the heart failure population, limiting the generalizability of our results. While we adjusted our analyses for age and sex, we chose not to adjust for differences in prevalence of comorbid conditions and cardiac risk factors between investigated groups, since a varying degree of involvement of non-cardiac mechanisms is inherent to the various HF groups. Apart from specific forms of cardiomyopathy, where HF may be isolated to cardiac manifestation, HF should always be regarded a multifactorial syndrome coexisting with systemic comorbidities. Thus, adjusting for such covariates may lead to overcorrection and inappropriate attenuation of the results. By only including HFrEF patients with ischemic etiology in our analysis, we aimed to improve the homogeneity of the proteomic profile in this group. Moreover, although each of the cohort studies followed their own protocols, the procedures for processing of the blood samples were similar, and proteomics measurements were performed according to the same method for both studies, in the same laboratory, and during the same timeframe. Second, SOMAscan is a generally highly specific and sensitive method for protein assessment that was assessed by independent studies^53^. However, some level of cross-reactivity with homologous proteins cannot be fully excluded. The results obtained with the aptamer-based SOMAscan platform do not provide exact quantification and do not fully correspond to other quantification methods. Our results should be further validated with absolute methods for any use in clinical practice. Finally, in our cohort, there was a low prevalence of atrial fibrillation in the HFpEF group. Patients included in the HELPFul study were referred to the cardiology policlinics by general practitioners for additional diagnostics, and diagnosed with HFpEF by experienced specialists. It is possible that other cardiac comorbidities were detected in the further diagnostic process in these patients, but were not yet known at the moment of inclusion.

## Conclusions

We found 2122 DEPs between iHFrEF and Stage A/Healthy and 52 DEPs between HFpEF and Stage A/Healthy. Of these 52 DEPs, 50 were also found in iHFrEF vs. Stage A/Healthy, leaving two proteins (SLITRK6 and NELL2) with significantly lower levels of expression in patients with HFpEF, but not iHFrEF. One other protein was expressed in the opposite direction between HFpEF and iHFrEF (MATN2). Moreover, we found 1462 DEPs between iHFrEF and HFpEF, out of which 108 were found uniquely between these groups. The analysis in men and women showed engagement of different mechanisms in development of HF. Altogether, the presence of overlapping DEPs in HFpEF and iHFrEF compared to Stage A/Healthy, underlines their involvement in HF irrespective of its underlying etiology. Conversely, presence of unique DEPs, found only between particular groups, contributes to the understanding of the differences in mechanisms between various types of HF.

## Methods

### Study design

We combined the data from two observational studies, which are described in detail elsewhere^54,55^.

The Bio-SHiFT study (“Serial Biomarker Measurements and New Echocardiographic Techniques in Chronic Heart Failure Patients Result in Tailored Prediction of Prognosis”; Clinicaltrials.gov: NCT01851538) is a prospective cohort study of stable patients with chronic HF, conducted in Erasmus MC, Rotterdam and Northwest Clinics, Alkmaar, Netherlands. In brief, the Bio-SHiFT study included 398 ambulatory adult patients if CHF had been diagnosed according to European Society Guidelines at least 3 months before, and the clinical course was currently stable (i.e., they had not been hospitalized for HF in the past 3 months). For the purposes of the current analysis data of the 170 patients with known ischemic etiology of HFrEF were used. We have previously examined the associations of circulating proteins with prognosis in the full Bio-SHiFT cohort, including proteomic analysis with the Olink panel^56^ and the SomaLogic panel^57,58^.

HELPFul (“Discovery of biomarkers for the presence and progression of left ventricular diastolic dysfunction and HEart faiLure with Preserved ejection Fraction in patients at risk for cardiovascular disease”; Dutch Trial Register: NTR6016) is a prospective cohort study of individuals referred by the general practitioner to a cardiology outpatient clinic, in Utrecht, the Netherlands. In the HELPful cohort, all patients aged 45 years and older, without previous cardiac interventions or congenital heart disease, who were referred by the general practitioner to the cardiology outpatient clinic were eligible for inclusion. Each week, on three out of four inclusion days, only patients with elevated LV filling pressures (defined as an E/e′ ≥ 8.0) were eligible for inclusion. On the fourth day, 25% of all patients attending that day were invited to participate, regardless of their echocardiography results. In the context of the HELPful study, two previous studies have been published, examining proteomics and HFpEF^59,60^.

Both studies were approved by the responsible medical ethics committees and conducted in accordance with the Declaration of Helsinki. The studies did not interfere with routine patient care. All participants have given informed consent. For the current analysis, we categorized patients into four groups: (i) Stage A/Healthy—Non-HF participants with normal filling pressures; (ii) Stage B—Non-HF participants with elevated filling pressures; (iii) iHFrEF; (iv) HFpEF.

### Blood sampling and proteomic analysis

Venous blood was collected from participants in both studies, processed and frozen following analogous protocols. Samples were aliquoted and frozen at − 80 °C within two hours from the moment of venepuncture. EDTA-plasma samples were sent (frozen, under controlled conditions) to SomaLogic (Boulder, Colorado) for SomaScan® V4 assay measurement, a platform for quantifying 5284 protein reagents, as described previously^4^. SomaScan proteomic analysis for both cohorts was performed in March 2020, following the same quality control procedure and acceptance criteria. SomaScan® was performed to identify differences in quantitative binding of proteins to aptamers, reflecting differences in protein expression. In total, 5284 modified aptamers were measured in both studies, of which aptamers with non-human or not validated targets were excluded. From aptamers targeting the same protein, those with the highest binding affinity were kept, while the remaining were excluded. Aptamers with a QC ratio outside of the expected range were excluded from the analyses (488 aptamers in HELPful and 610 aptamers in the BioSHiFT study, respectively). Hence, after filtering for abovementioned criteria, total of 4210 aptamers were available for the analysis.

### Statistical analysis

Data from both cohorts were merged and clinical variables were aligned where necessary. Baseline characteristics were compared between the groups by means of the independent samples t-test for normally distributed continuous variables, Mann–Whitney *U* test for non-normally distributed continuous variables, and Chi-squared test or Fisher's exact test for categorical variables, where appropriate. Proteomics data were log transformed and subsequently, the Z-score was calculated.

We performed age and sex corrected multinominal regression analysis to examine the associations between circulating proteins and heart failure group (iHFrEF, HFpEF, Stage B, Stage A/Healthy). Circulating proteins were entered as independent variables, and group membership was the dependent variable. We calculated the odds ratios and 95% confidence intervals for each group, compared to the reference group. First, Stage A/Healthy was used as the reference group. Subsequently, to further investigate differences between iHFrEF and HFpEF and Stage B and HFpEF, the HFpEF group was used as the reference. We corrected for multiple testing using the Benjamini–Hochberg method (False Discovery Rate < 0.05). We repeated all analyses in men and women separately, with adjustment for age.

All the DEPs that reached statistical significance were analysed using Protein Set Enrichment Analysis in ToppGene^9^, if the total number of DEPs allowed for this. DEPs detected between groups were used as the Enrichment Protein Set; all examined aptamers were used as the Background Set.

Two-sided p-values < 0.05 were considered statistically significant for analyses other than the multinomial regression. R Studio version 4.0.3 (https://cran.r-project.org/bin/windows/base/old/4.0.3) was used for all analyses, including packages nnet and ggplot.

### Supplementary Information

Supplementary Figure S1.

Supplementary Figure S2.

Supplementary Figure S3.

Supplementary Table S1.

Supplementary Table S2.

Supplementary Table S3.

Supplementary Table S4.

## Data Availability

The data underlying this article will be shared on reasonable request to the corresponding author.
