# Supplementary material for: Identifying plasma proteomic signatures from health to heart failure, across the ejection fraction spectrum
Source: Sci Rep. 2024 Jun 27;14:14871. doi: 10.1038/s41598-024-65667-0 (PMC11211454; doi:10.1038/s41598-024-65667-0)

Plasma Proteomic Signatures from Health to Heart Failure: towards identifying specific proteomic patterns across the heart failure spectrum

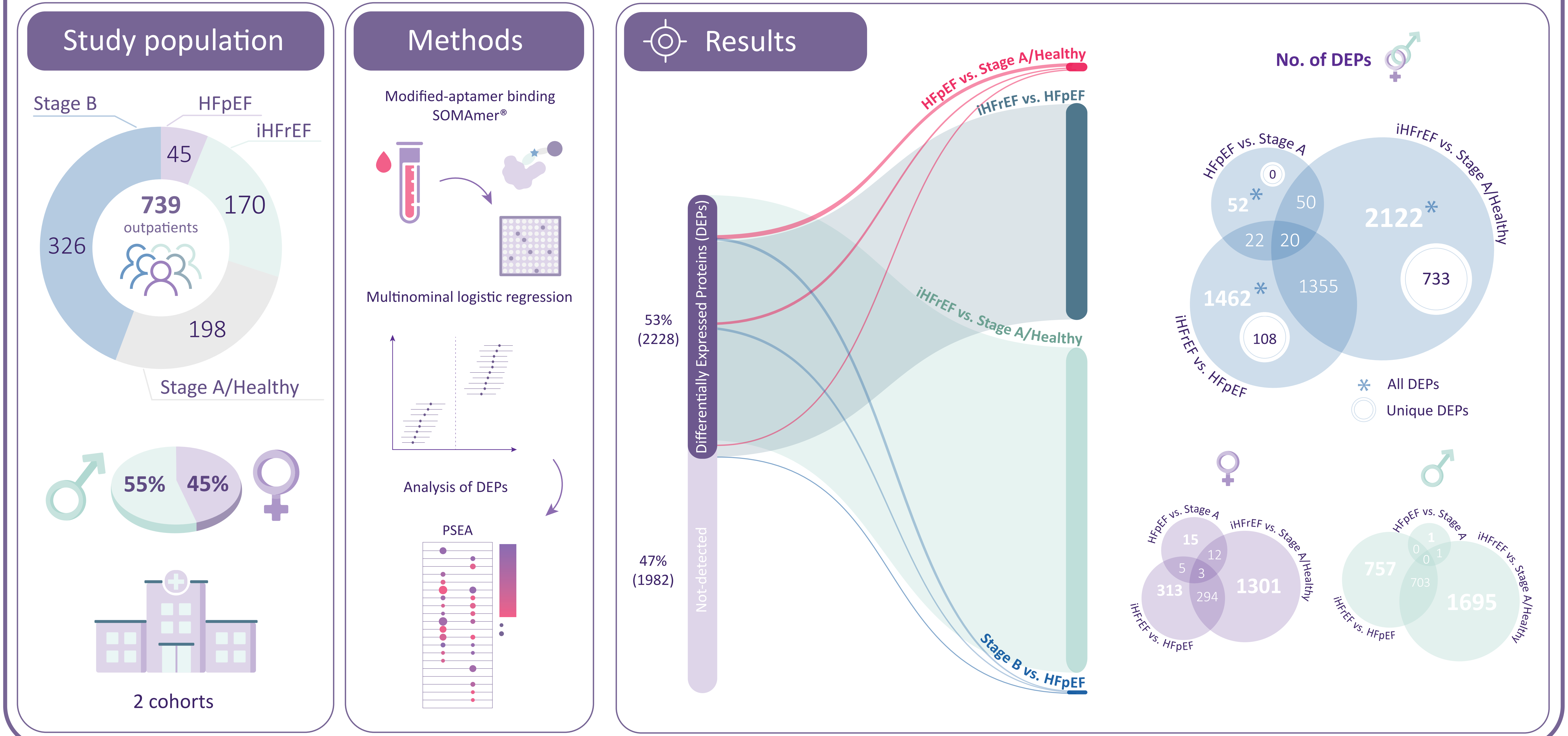

Supplement: Supplementary file 1 — Supplementary Figure S1. [file 41598_2024_65667_MOESM1_ESM.pdf]
