# Supplementary material for: Identifying plasma proteomic signatures from health to heart failure, across the ejection fraction spectrum
Source: Sci Rep. 2024 Jun 27;14:14871. doi: 10.1038/s41598-024-65667-0 (PMC11211454; doi:10.1038/s41598-024-65667-0)
Supplement: Supplementary file 2 — Supplementary Figure S2. [file 41598_2024_65667_MOESM2_ESM.pdf]

Figure S1. Top 25 DEPs with the highest and the lowest levels found in iHFrEF vs. Stage A/Healthy and iHFrEF vs. HFpEF in women (A, B, C, D) and men (E, F, G, H). All DEPs found in HFpEF vs. Stage A/Healthy in women (I).

A. Top 25 DEPs with the highest levels in iHFrEF, vs. Stage A/Healthy in women

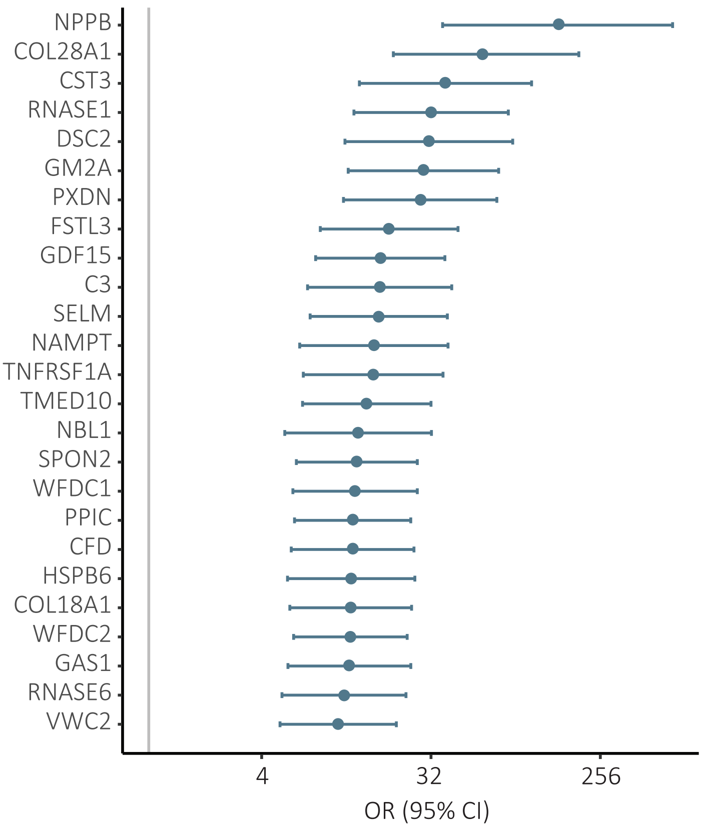

B. Top 25 DEPs with the lowest levels in iHFrEF, vs. Stage A/Healthy in women

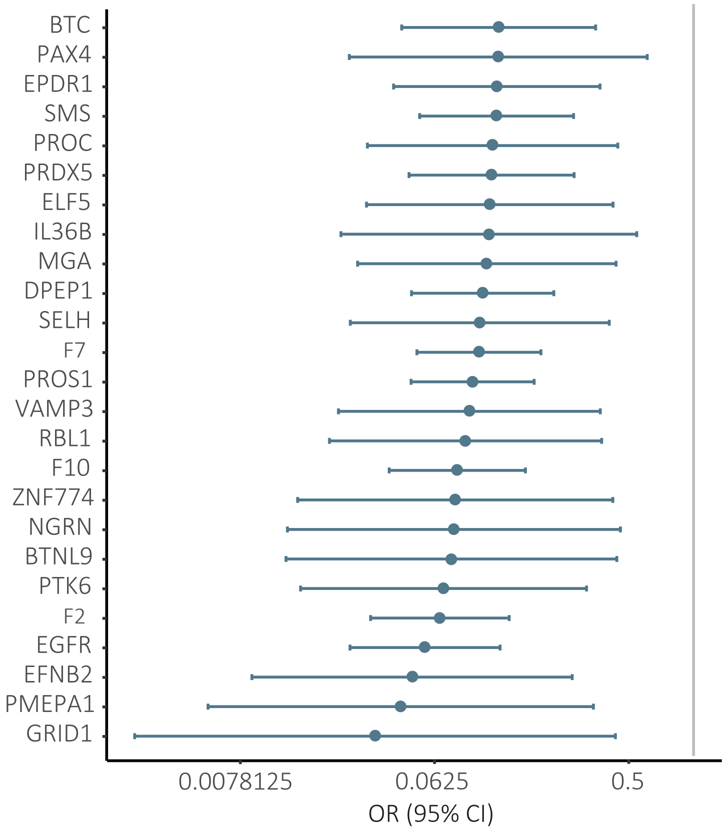

C. Top 25 DEPs with the highest levels in iHFrEF, vs. HFpEF in women

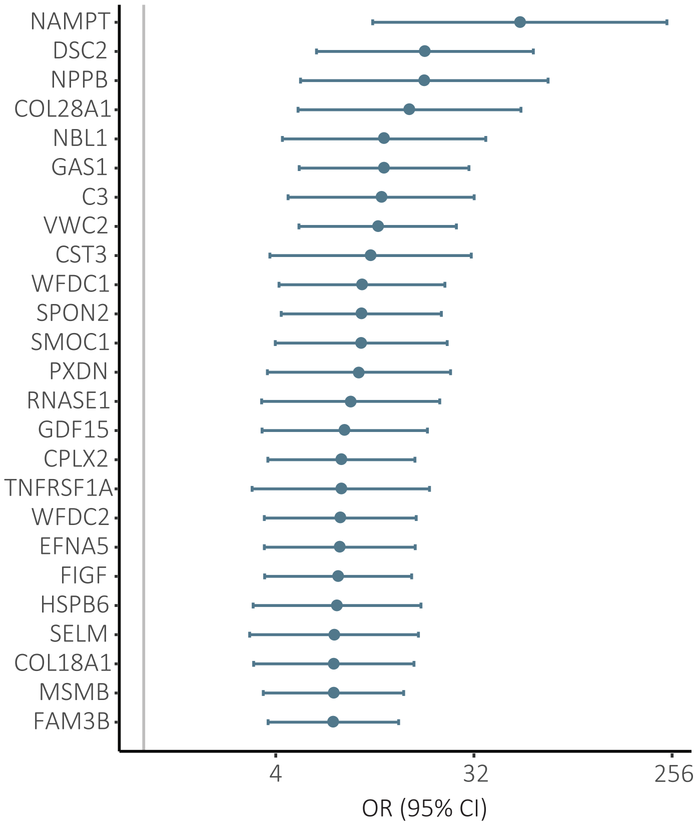

D. Top 25 DEPs with the lowest levels in iHFrEF, vs. HFpEF in women

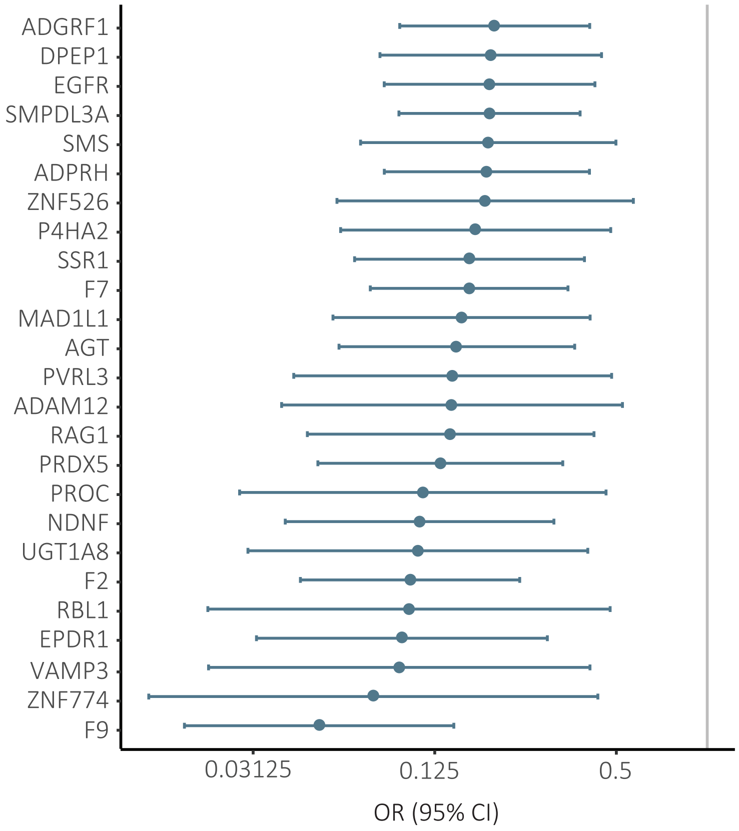

E. Top 25 DEPs with the highest levels in iHFrEF, vs. Stage A/Healthy in men

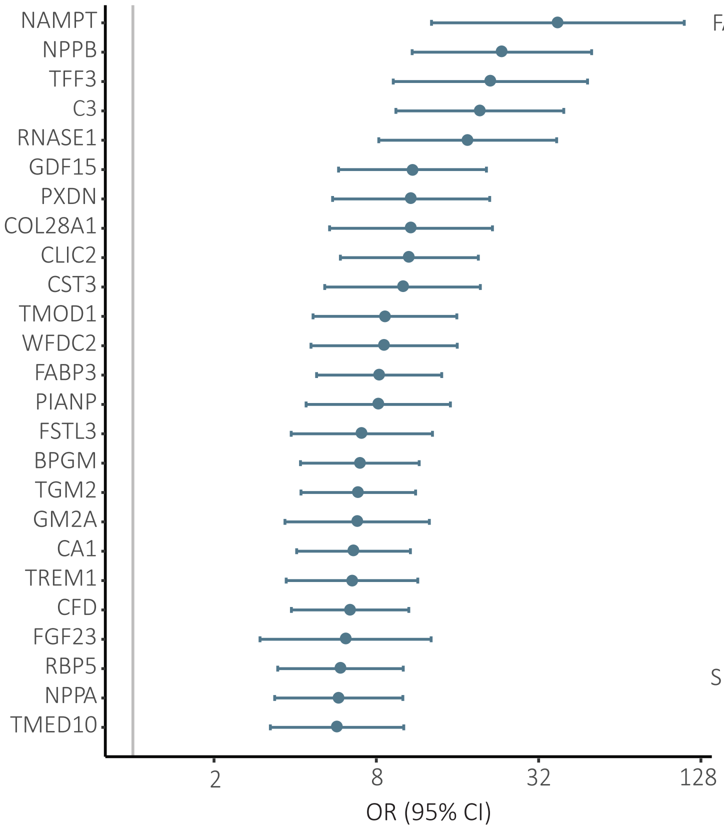

F. Top 25 DEPs with the lowest levels in iHFrEF, vs. Stage A/Healthy in men

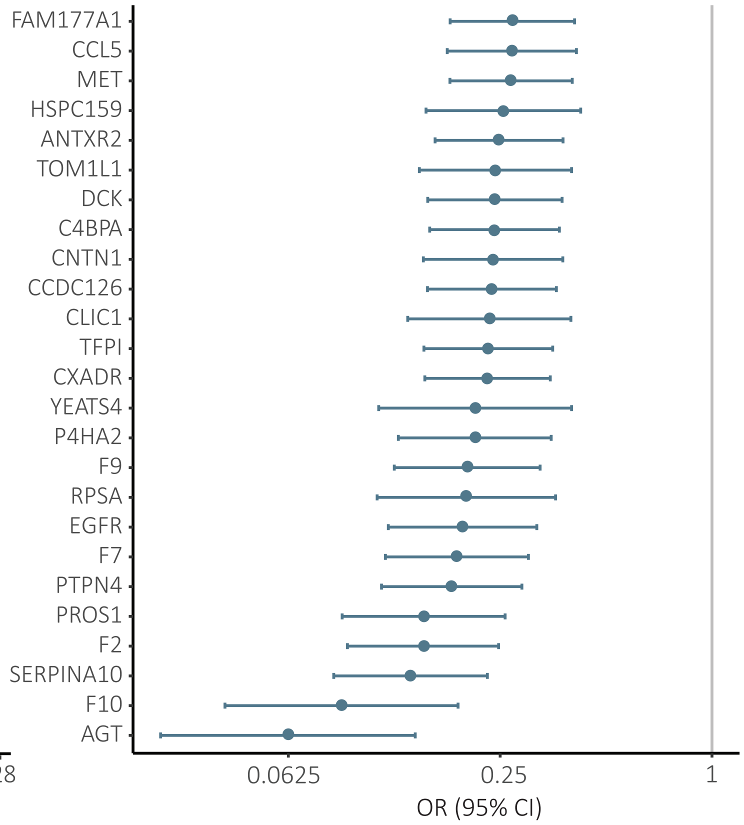

G. Top 25 DEPs with the highest levels in iHFrEF, vs. HFpEF in men

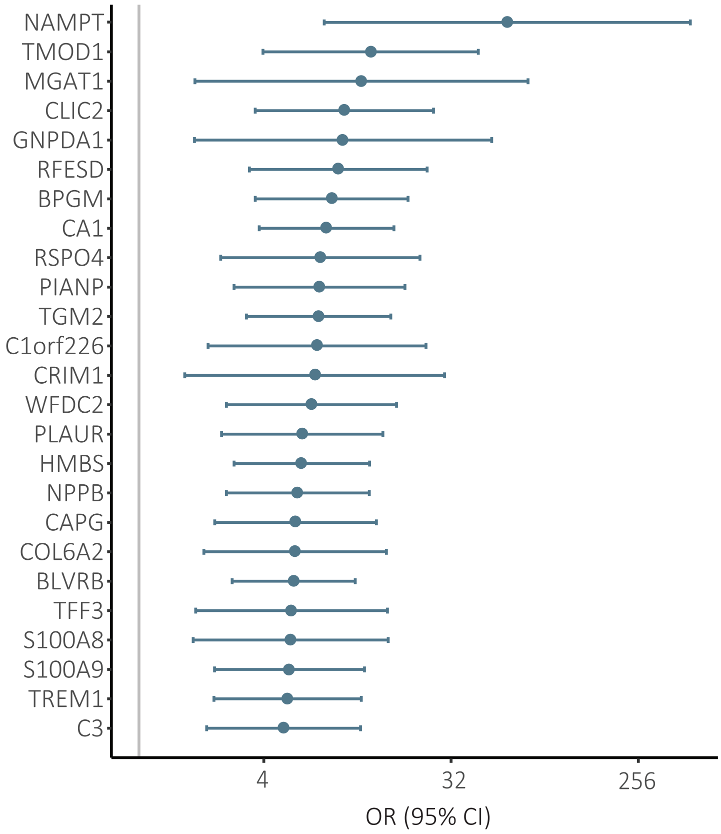

H. Top 25 DEPs with the lowest levels in iHFrEF, vs. HFpEF in men

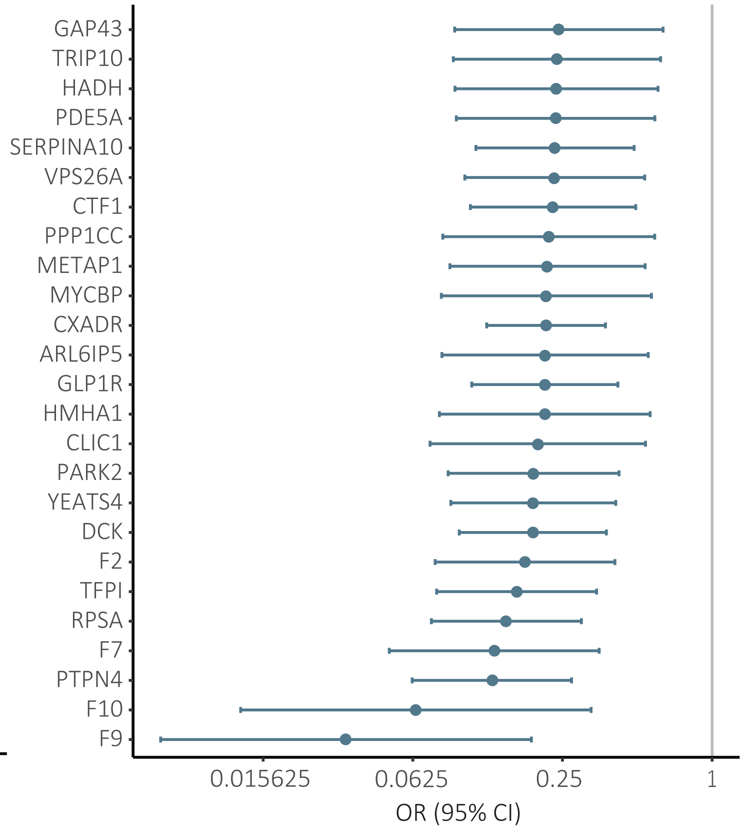

I. All DEPs in HFpEF vs. Stage A/Healthy in women

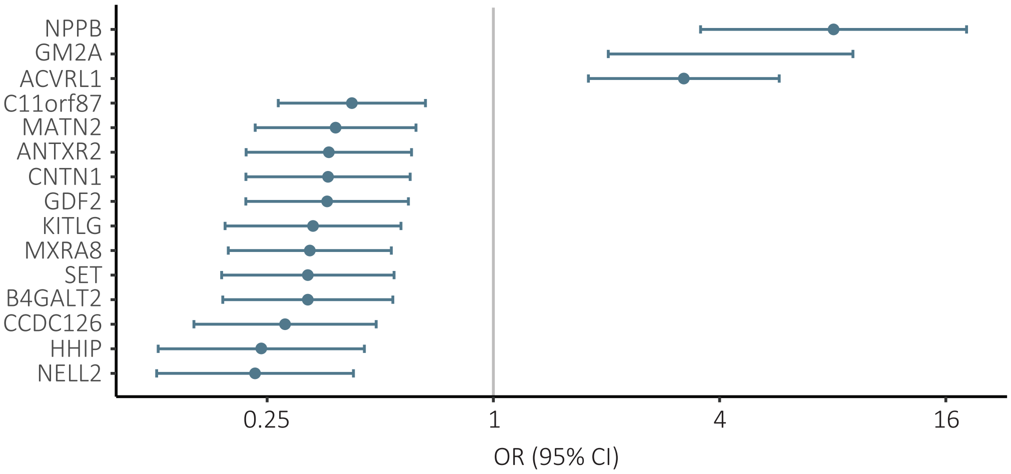

In this figures we plotted the single-protein associations (adjusted for age) with iHFrEF compared to Stage A/Healthy as the reference group (Figure S1: A, B in women; E, F in men) and HFpEF as the reference group (Figure S1: C, D in women; G,H in men) and HFpEF vs. Stage A/Healthy in women (Figure S1. I), resulting from the multinominal regression analysis. The HF group was used as the dependent variable and the protein level as independent variable. Top 25 upregulated and downregulated DEPs that showed statistically significant associations after adjustment for multiple testing are presented. All DEPs found between HFpEF vs. Stage A/Healthy in women are presented. X-axis is on the log2 scale.
