# Supplementary material for: Identifying plasma proteomic signatures from health to heart failure, across the ejection fraction spectrum
Source: Sci Rep. 2024 Jun 27;14:14871. doi: 10.1038/s41598-024-65667-0 (PMC11211454; doi:10.1038/s41598-024-65667-0)
Supplement: Supplementary file 3 — Supplementary Figure S3. [file 41598_2024_65667_MOESM3_ESM.pdf]

Figure S2 Protein Set Enrichment Analysis of all significant differentially associated proteins (DEPs) between iHFrEF vs. HFpEF and iHFrEF vs Stage A/Healthy.

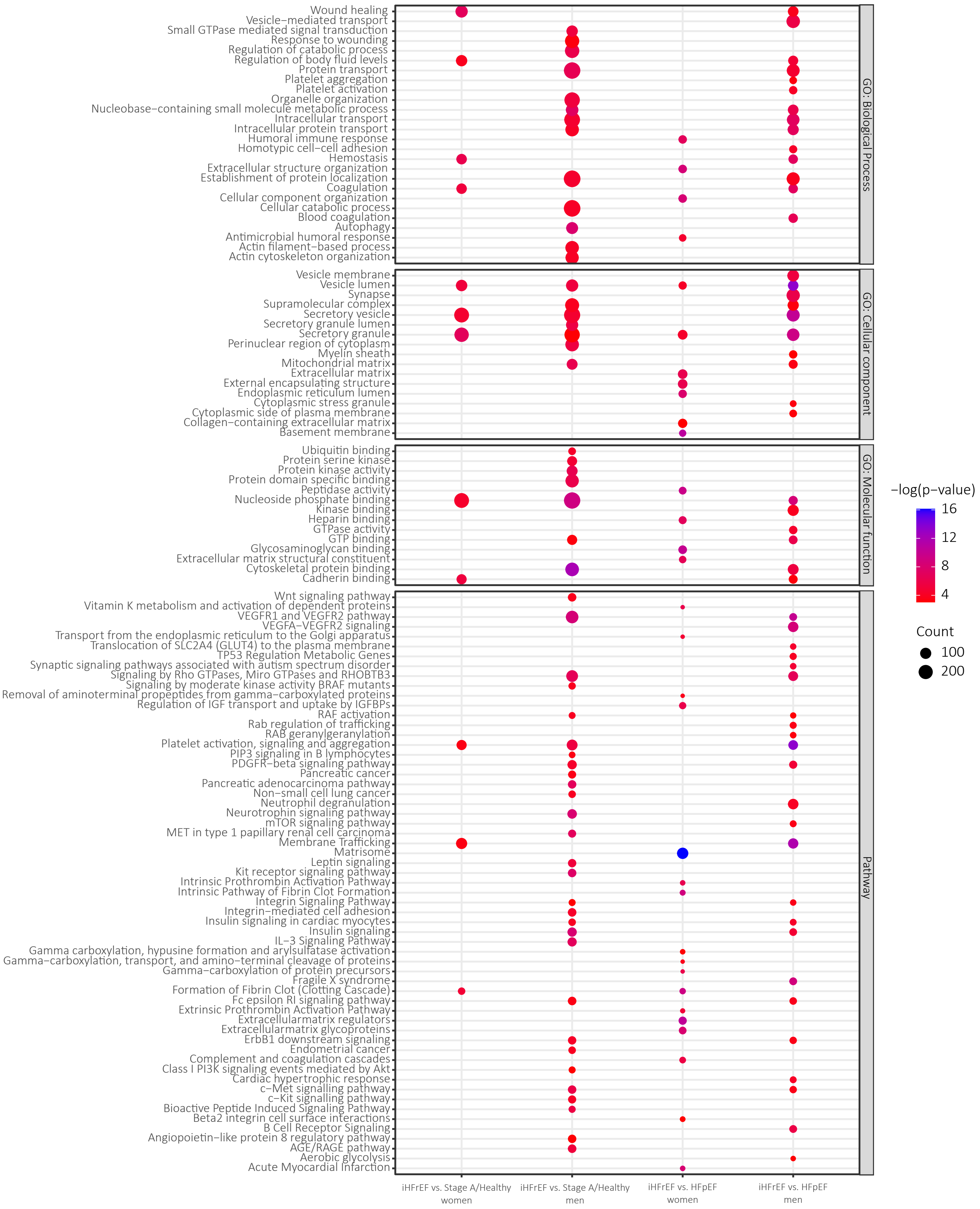

In this figure the colour of the dot represents the magnitude of the p-value. The size ("Count") represents how many of the included proteins are present in the mechanism or process.
